# Validity of self-measured waist circumference in adults at risk of type 2 diabetes and cardiovascular disease

**DOI:** 10.1186/s12916-014-0170-x

**Published:** 2014-10-02

**Authors:** Ana María Contardo Ayala, Giel Nijpels, Jeroen Lakerveld

**Affiliations:** Department of General Practice and Elderly Care Medicine, EMGO Institute for Health and Care Research, VU University Medical Center, van der Boechorststraat 7, 1081 BT Amsterdam, The Netherlands

**Keywords:** Anthropometric measurements, Waist circumference, Screening, Obesity, Type 2 diabetes mellitus, Cardiovascular disease, Population studies, Prevention

## Abstract

**Background:**

Waist circumference (WC) is used to indirectly measure abdominal adipose tissue and the associated risk of type 2 diabetes mellitus (T2DM) and cardiovascular disease (CVD). Because of its easy implementation and low cost, self-measured WC is commonly used as a screening tool. However, discrepancies between self-measured and objectively measured WC may result in misclassification of individuals when using established cut-off values. The aim of this study was to determine the accuracy of self-measured WC in adults at risk of T2DM and/or CVD, and to determine the anthropometric, demographic and behavioural characteristics associated with bias in self-measured WC.

**Methods:**

Self-measured and objectively measured WC was obtained from 622 participants (58.4% female; mean age 43.4 ± 5.3 years) in the Hoorn Prevention Study. The associations of gender, age, educational level, body mass index, smoking status, dietary habits, physical activity and sedentary behaviour with the discrepancies between self-measured and objectively measured WC were analysed using independents *t*-test and one-way ANOVA. Bland-Altman plots were used to plot the agreement between the two measures.

**Results:**

On average, self-measured WC was overestimated by 5.98 ± 4.82 cm (*P* < 0.001). Overestimation was consistent across all subgroups, but was more pronounced in those who were younger and those with lower educational attainment.

**Conclusions:**

The results support self-measured WC as a useful tool for large-scale populations and epidemiological studies when objective measurement is not feasible, but overestimation should be taken into account when screening adults at risk of T2DM and/or CVD.

## Background

Abdominal adipose tissue, in particular visceral adipose tissue, is associated with increased risk of developing chronic diseases such as type 2 diabetes mellitus (T2DM) and cardiovascular disease (CVD), independently of whole body adiposity [1-5]. Because of its simplicity, cost-effectiveness and non-invasive characteristics, the waist circumference (WC) measure is the most common choice in clinical settings to estimate visceral adipose tissue. Despite being an indirect anthropometric measure, WC is also widely used to categorise populations at risk of T2DM and CVD in epidemiological studies [1,3-5]. WC has been shown to be more sensitive than body mass index (BMI) for identifying T2DM and CVD risk, as BMI has shown to be less able to differentiate than WC between adipose tissue and lean mass [6-8]. As an initial screening tool, WC is able to identify individuals that may need further assessment, and thus can assist in prioritising and targeting health actions in specific populations [7,9].

In epidemiological studies and health promotion programmes, self-measured WC is often applied by providing individuals with a measuring tape and a recording form. However, and in contrast to self-measured BMI, the accuracy of self-measured WC is less well established [10-18]. Furthermore, contradictory results have raised doubts about the validity of self-measured WC [10,12,14-19]. As self-measured WC is widely used in large-scale epidemiological studies as a primary screening tool, an overestimation of WC may unnecessarily increase the demand on healthcare systems, whereas an underestimation may lead to misclassification of candidates for preventive or treatment programmes [1]. It is therefore important to gain a better understanding of the accuracy of self-measured WC, and the characteristics associated with discrepancies between self-measured and objectively measured WC.

To date, the validity of self-measured WC has not been established for populations that are explicitly characterised by their increased estimated risk of developing T2DM and CVD. Moreover, to our knowledge, no study has explored which individual-level characteristics, such as dietary behaviour, physical activity and sedentary behaviours, relate to bias in self-measured WC. The aim of this study was to assess the accuracy of self-measured WC in adults at risk of T2DM and/or CVD, and to explore which anthropometric, demographic and behavioural characteristics are associated with the accuracy of self-measured WC.

## Methods

### Study population

The study was approved by the Medical Ethics Committee of the VU University Medical Center in Amsterdam, and all participants provided written informed consent.

For this validation study, data was used of the Hoorn Prevention Study, described in detail elsewhere [20-22]. In brief, the Hoorn Prevention Study is a randomised control trial aiming to investigate the effects of a theory-based lifestyle intervention on targeting the estimated risk of developing T2DM and CVD mortality in adults at risk.

For this validation study, the study population (n = 8,193, age 30 to 50 years) received an invitation package that included a tape measure and instructions for self-measurement of their WC. Of the 3,587 respondents (43.8%), 921 were invited for further screening because their self-reported WC exceeded the pre-set cut-off score of self-measured WC (≥101 cm for men and ≥87 cm for women). Of this sample, 772 individuals gave written informed consent and visited the research centre for objective measurements. The 9-year risk of developing T2DM risk was calculated for all 772 participants according to the procedure described in the diabetes risk formula of the Atherosclerosis Risk In Communities (ARIC) Study [23], and the 10-year risk of a fatal CVD was estimated using the Systematic Coronary Risk Evaluation (SCORE) project [24]. After this step, another 150 people were excluded (140 had a risk lower than 10% in both scores, and 10 had undiagnosed T2DM). The final study population comprised 622 participants.

### Data collection

For the purpose of the current study, anthropometric, demographic and behavioural characteristics were extracted from self-reported questionnaires. The objectively measured anthropometric data were recorded in the research centre.

#### Anthropometric characteristics

Self-measured waist circumference was obtained from a form that was mailed to all participants together with a measuring tape and detailed instructions. These instructions specified that the circumference of the (bare) belly should be measured just above the navel, as indicated in a silhouette picture that was displayed next to the instructions.

Objectively measured waist circumference was obtained by trained medical research assistants. This was done locating the tape measure midway between the lowest rib margin and the iliac crest. Two measurements rounded to the nearest 0.5 cm were recorded; if the differences between the measurements was greater than 1 cm, a third measurement was performed, and the mean of the two closest measurements was calculated.

Weight was measured rounding to the nearest 0.5 kg and height was measured rounding to the nearest 0.1 cm (when wearing light clothes and no shoes). BMI was calculated as weight divided by height squared, and was stratified for the analysis into three categories: normal, overweight and obese, also according to the WHO guidelines [25].

#### Demographic characteristics

Age, gender and level of education were obtained from the forms filled out by the participants. Age groups (younger and older) were set by dividing the whole sample into two equally sized groups. Level of education was defined as ‘primary or lower’, ‘secondary’ or ‘college/university’.

#### Behavioural characteristics

Dietary behaviour*:* Participants were divided into those who met the national recommendation of at least two pieces of fruit and 200 g of vegetable intake per day or not [26], using an eight-item food frequency questionnaire.

Smoking behaviour was determined using the WHO guideline for smoking status (smoke every day/occasionally/never smoke) [27]*.* However, for the purpose of this study, we grouped the ‘smoke every day’ and ‘smoke occasionally’ groups into one.

Physical activity behaviour was assessed using the Short Questionnaire to Assess Health Enhancing Physical Activity (SQUASH), which enables a relative valid estimation of physical activity level in adults [28]. Participants were divided into those who met the national recommendation for physical activity of more than 30 minutes of moderate-intensity physical activity (for example, brisk walking) at least 5 days/week, or not.

Sedentary behaviour was assessed using a sub-scale of the Activity Questionnaire for Adolescents and Adults (AQuAA) [29]. We classified participants as sedentary when sedentary time, defined as activities with energy expenditure of under 2 Metabolic Equivalent of Task (1 MET = 3.5 ml O_2_ · kg^−1^ · min^−1^ ) during leisure time exceeded 2 hours per day [30].

### Statistical analysis

The strength of the relationship between self-measured and objectively measured WC was investigated using the Pearson correlation coefficient. The agreement between the measurements was plotted using Bland-Altman plots [31,32], with the difference between the two measurements plotted against the mean of the two measurements. Limits of agreement were calculated as the mean difference ±1.96 standard deviations (SD).

To identify variables that explained differences in the objectively measured and self-measured WC, independent *t*–tests were carried out to assess the statistically significant difference within subgroups divided by age, gender, dietary behaviour, smoking behaviour, physical activity and sedentary behaviour. Furthermore, one-way ANOVA was used to determine the association of the mean difference between self-measured and objectively measured WC with BMI and level of education categories. All analyses were performed using IBM-SPSS Statistics for Windows, version 20.0.

## Results

**Figure 1:**
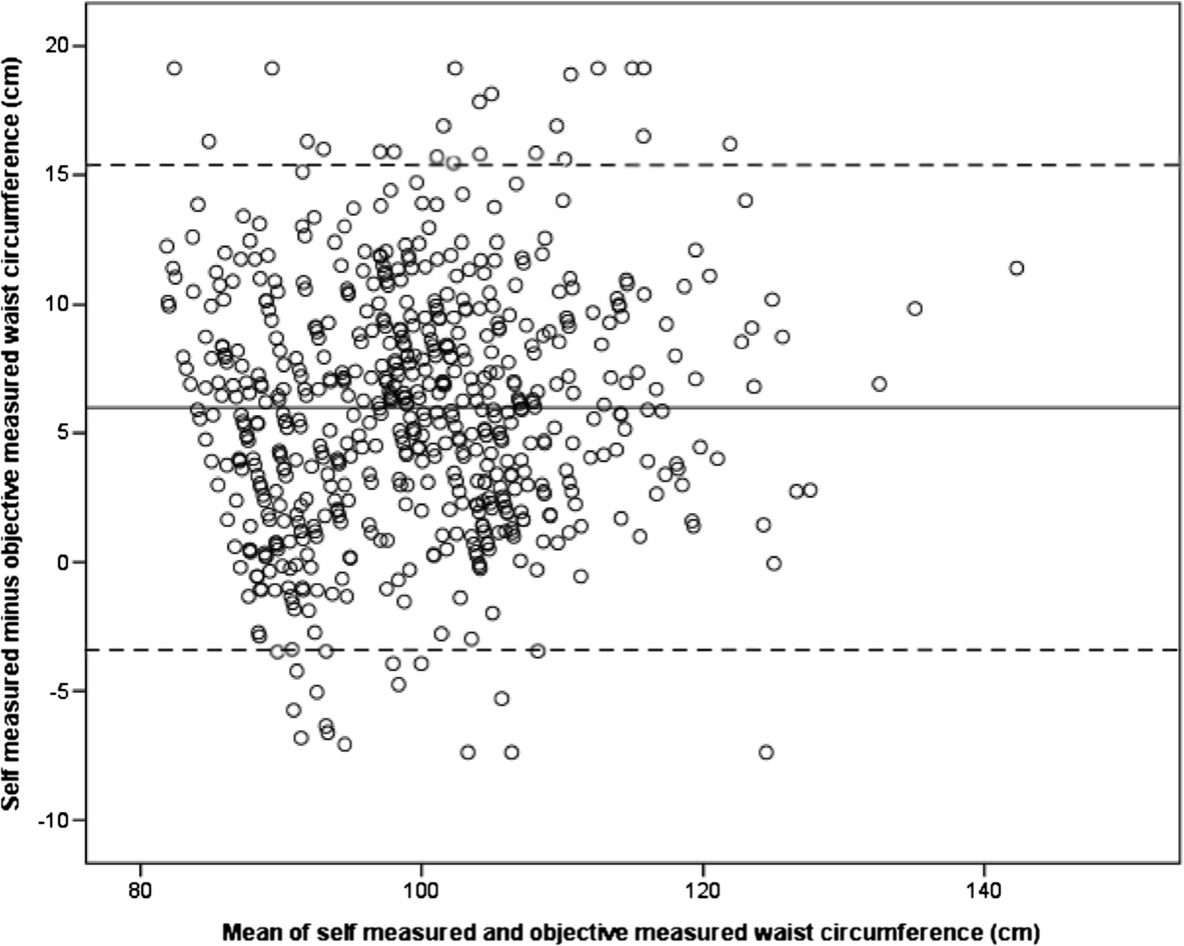
**Bland-Altman plot of the difference between self-measured and objectively measured waist circumference plotted against the mean.** The solid line represents the mean difference between the objectively and self-measured waist circumference (5.98 cm), and the dashed lines represent the 95% confidence interval for agreement (15.42 to −3.46).

Objectively measured WC was obtained for all participants, but self-measured WC was missing for five individuals. Table 1 shows the mean values of objectively measured and self-measured WC and the mean differences between these two measures. Although a strong correlation was found between objectively and self-measured WC (*r* = 0.87, *n* = 617, *P* < 0.001), 90.6% of the participants overestimated their WC by a mean of 5.98 cm ± 4.82 (*P* < 0.001). Figure 1 depicts the extent of misreporting of WC (Bland-Altman plot). The differences between self-measured and objectively measured WC ranged from 15.42 cm (overestimation) to −3.46 cm (underestimation).

**Table 1:** **Mean (SD) self-measured waist circumference and objectively measured waist circumference and their mean differences (95% CI) stratified by anthropometric, demographic and behavioural characteristics**

**Categories**	***n***	**SMWC**		**OMWC**		**SMWC- OMWC mean (95%** **CI)** ^**a**^			***P*** **-value** ^**b**^
		**Mean**	**SD**	**Mean**	**SD**	**Mean**	**Low**	**High**	
Overall	617	102.6	10.1	96.6	9.7	6.0	6.4	5.6	<0.01^b^
Age^c^									
Younger	309	103.9	10.6	97.4	9.8	6.4	6.9	5.9	0.02^b^
Older	308	101.3	9.4	95.8	9.6	5.5	6.1	5.0	
Education									
Primary	201	103.5	11.2	96.9	11.1	6.6	7.3	5.9	0.03^b^
Secondary	285	102.7	9.8	96.8	9.3	5.9	6.5	5.4	
College	128	100.8	8.7	95.6	8.2	5.2	6.0	4.4	
Body mass index^d^									
Normal	84	93.7	6.1	87.3	6.0	6.4	7.5	5.3	0.56
Overweight	333	100.3	7.6	94.5	7.3	5.8	6.3	5.3	
Obese	200	110.1	10.3	104.0	9.4	6.1	6.8	5.4	
Dietary behaviour^e^									
Yes	47	99.3	9.1	94.1	9.1	5.2	6.6	3.8	0.23
No	531	102.7	10.1	96.7	9.7	6.1	6.5	5.7	
Smoking behaviour^f^									
Yes	126	104.3	11.5	97.9	10.7	6.4	7.1	5.6	0.33
No	487	102.1	9.7	96.2	9.4	5.9	6.3	5.5	
Physical activity^g^									
Yes	383	101.8	9.8	96.1	9.4	5.8	6.3	5.3	0.26
No	230	103.8	10.6	97.4	10.3	6.4	7.0	5.8	
Sedentary behaviour^h^									
Yes	542	102.8	10.1	96.8	9.8	6.0	6.4	5.6	0.74
No	69	100.4	10.0	94.5	9.0	5.8	6.9	4.7	

*Abbreviations*: OMWC, objectively measured waist circumference; SMWC, self-measured waist circumference.

^a^Difference calculated as self-measured minus objectively WC values, note that negative and positive values indicates under and over -estimation, respectively.

^b^Significant differences (*P* < 0.05).

^c^Age groups: younger ≤44.07 years and older >44.07 years.

^d^Body mass index: normal <25.0 kg/m^2^, overweight ≥25.0 to <29.9 kg/m^2^, obese ≥30.0 kg/m^2^.

^e^Diet: Yes: meeting recommendations of at least two pieces of fruit and 200 g of vegetables intake per day; No: not meeting these recommendations.

^f^Smoking behaviour: Yes: current smoker: No: not current smoker.

^g^Physical activity: Yes: meeting the national recommendations of being physically active (≥30 minutes moderate-intensity physical activity), No: not meeting these recommendations.

^h^Sedentary Behaviour: Yes: sedentary (≥2 hours per day activities with an energy expenditure of under 2 METs); No: not meeting these recommendations.

Compared with the older participants (47.9 ± 2.0 years), the younger (39 ± 3.4 years) group overestimated WC by more (5.5 ± 4.9 cm and 6.4 ± 4.6 cm, respectively) (*P* = 0.018). Participants in the lowest educational level group overestimated their WC significantly more than the group with the highest education (6.6 ± 5 cm and 5.2 ± 4.6 cm, respectively) (*P* = 0.028).

## Discussion

In this study, we assessed the validity of self-measured WC, and whether anthropometric, demographic and behavioural characteristics were associated with differences between self-measured and objectively measured WC. Overall, the vast majority of the individuals overestimated their WC. We found that age and education level were associated with a higher discrepancy between self-measured and objective measured WC, with those who where younger and less educated overestimating the most. Self-measured WC was not found to be affected by other variables such as gender, BMI, dietary behaviour, smoking status, physical activity or sedentary behaviour.

In agreement with our findings, several previous studies have reported an overestimation of WC [10,15,16,18,33,34], but other studies have found an underestimation of self-measured WC compared with objectively measured WC [11-14,17,19,35,36]. Higher values of BMI [13,14,17] were associated with a higher degree of underestimation, with females underestimating more than males [17]. The inconsistency of previous findings may be explained by the heterogeneity of populations under study (for example, children, older adults with heart failure). Interestingly, we found an effect of educational attainment on WC overestimation, which has not been reported previously.

To our knowledge, no published literature has reported on the effect of dietary, physical activity and sedentary behaviours as a potential source of bias in WC self-measurements. Understanding potential lifestyle-related behavioural biases is important as these behaviours are associated with an increased BMI and WC [37], which, in turn, have been shown to bias self-measured WC [13,14]. However, in the current study, no significant overestimation of WC was found for those engaged in unhealthy behaviours compared with those who had a healthier lifestyle.

A potential limitation should be taken into account. We only had objective WC measurements from those who self-reported a WC above our pre-set threshold. As a result, respondents who underestimated their self-measured WC may have been missed in the analyses for the current study, as they did not pass the initial screening step, and they could also have missed out on the intervention.

## Conclusion

In conclusion, we found a systematic overestimation of WC in a Dutch adult population at risk of T2DM and/or CVD. This overestimation was relatively higher in those who were younger and those who had primary level education or less. The present study supports the utilisation of self-measured WC for screening in preventive/treatment programmes and epidemiological studies when objective measurement is not feasible. This measure is considered a useful and inexpensive clinical tool that can easily be implemented to routine health assessments and health promotion, and as inclusion criteria for epidemiological studies. We would, however, advise using a slightly lower cut-off score, considering the systematic overestimation of self-reported WC.

## Abbreviations

BMI: Body mass index
CVD: Cardiovascular disease
T2DM: Type 2 diabetes mellitus
WC: Waist circumference

## References

[1] Hernan A, Philpot B, Janus ED, Dunbar JA (2012). Recruitment into diabetes prevention programs: what is the impact of errors in self-reported measures of obesity?. BMC Public Health.

[2] Poirier P, Despres J-P (2003). Waist circumference, visceral obesity, and cardiovascular risk. J Cardiopulm Rehabil.

[3] Siren R, Eriksson JG, Vanhanen H (2012). Waist circumference a good indicator of future risk for type 2 diabetes and cardiovascular disease. BMC Public Health.

[4] Snijder MB, van Dam RM, Visser M, Seidell JC (2006). What aspects of body fat are particularly hazardous and how do we measure them?. Int J Epidemiol.

[5] Visscher TLS, Seidell JC (2001). The public health impact of obesity. Annu Rev Public Health.

[6] Janssen I, Katzmarzyk PT, Ross R (2004). Waist circumference and not body mass index explains obesity-related health risk. Am J Clin Nutr.

[7] Korhonen PE, Jaatinen PT, Aarnio PT, Kantola IM, Saaresranta T (2009). Waist circumference home measurement—a device to find out patients in cardiovascular risk. Eur J Public Health.

[8] Lean MEJ, Han TS, Morrison CE (1995). Waist circumference as a measure for indicating need for weight management. Br Med J.

[9] Klein S, Alison DB, Heymsfield SB, Kelley DE, Leibel RL, Nonas C, Kahn R (2007). Waist Circumference and Cardiometabolic Risk: A Consensus Statement from Shaping America’s Health: Association for Weight Management and Obesity Prevention; NAASO, The Obesity Society; the American Society for Nutrition; and the American Diabetes Association. Obesity.

[10] Dekkers JC, van Wier MF, Hendriksen IJM, Twisk JWR, van Mechelen W (2008). Accuracy of self-reported body weight, height and waist circumference in a Dutch overweight working population. BMC Med Res Methodol.

[11] Lim LLY, Seubsman SA, Sleigh A, Bain C (2012). Validity of self-reported abdominal obesity in Thai adults: a comparison of waist circumference, waist-to-hip ratio and waist-to-stature ratio. Nutr Metab Cardiovasc Dis.

[12] Roberts CA, Wilder LB, Jackson RT, Moy TF, Becker DM (1997). Accuracy of self-measurement of waist and hip circumference in men and women. J Am Diet Assoc.

[13] Spencer EA, Roddam AW, Key TJ (2004). Accuracy of self-reported waist and hip measurements in 4492 EPIC-Oxford participants. Public Health Nutr.

[14] Bigaard J, Spanggaard I, Thomsen BL, Overvad K, Tjønneland A (2005). Self-Reported and technician-measured waist circumferences differ in middle-aged men and women. J Nutr.

[15] Chan NPT, Choi KC, Nelson EAS, Sung RYT, Chan JCN, Kong APS (2012). Self-reported waist circumference: a screening tool for classifying children with overweight/obesity and cardiometabolic risk factor clustering. Pediatr Obes.

[16] Prince SA, Janssen I, Tranmer JE (2008). Self-measured waist circumference in older patients with heart failure - A study of validity and reliability using a MyoTape (R). J Cardiopulm Rehabil Prev.

[17] Park JY, Mitrou PN, Keogh RH, Luben RN, Wareham NJ, Khaw KT (2011). Effects of body size and sociodemographic characteristics on differences between self-reported and measured anthropometric data in middle-aged men and women: the EPIC-Norfolk study. Eur J Clin Nutr.

[18] den Engelsen C, van den Donk M, Gorter KJ, Salome PL, Bobbink IW, Rutten GE (2010). Detection of metabolic syndrome by self-measurement of waist circumference. Ned Tijdschr Geneeskd.

[19] Han TS, Lean MEJ (1998). Self-reported waist circumference compared with the ‘Waist Watcher’ tape-measure to identify individuals at increased health risk through intra-abdominal fat accumulation. Br J Nutr.

[20] Lakerveld J, Bot S, Chinapaw M, van Tulder M, Kingo L, Nijpels G (2012). Process evaluation of a lifestyle intervention to prevent diabetes and cardiovascular diseases in primary care. Health Promot Pract.

[21] Lakerveld J, Bot SDM, van der Ploeg HP, Nijpels G (2013). The effects of a lifestyle intervention on leisure-time sedentary behaviors in adults at risk: The Hoorn Prevention Study, a randomized controlled trial. Prev Med.

[22] Lakerveld J, Bot S, Chinapaw M, van Tulder M, Kostense P, Dekker J, Nijpels G (2013). Motivational interviewing and problem solving treatment to reduce type 2 diabetes and cardiovascular disease risk in real life: a randomized controlled trial. Int J Behav Nutr Phys Act.

[23] Schmidt M, Duncan B, Bang H, Pankow J, Ballantyne C, Golden S, Folsom A, Chambless L (2005). Identifying individuals at high risk for diabetes - The Atherosclerosis Risk in Communities study. Diabetes Care.

[24] Conroy RM, Pyörälä K, Fitzgerald AP, Sans S, Menotti A, De Backer G, De Bacquer D, Ducimetière P, Jousilahti P, Keil U, Njølstad I, Oganov RG, Thomsen T, Tunstall-Pedoe H, Tverdal A, Wedel H, Whincup P, Wilhelmsen L, Graham IM, SCORE project group (2003). Estimation of ten-year risk of fatal cardiovascular disease in Europe: the SCORE project. Eur Heart J.

[25] World Health Organization (2000). Obesity: Preventing and Managing the Global Epidemic. Report of a WHO Consultation.

[26] Bogers RP, van Assema P, Kester ADM, Westerterp KR, Dagnelie PC (2004). Reproducibility, validity, and responsiveness to change of a short questionnaire for measuring fruit and vegetable intake. Am J Epidemiol.

[27] World Health Organization (1998). Guidelines for Controlling and Monitoring the Tobacco Epidemic.

[28] Wendel-Vos GCW, Schuit AJ, Saris WHM, Kromhout D (2003). Reproducibility and relative validity of the short questionnaire to assess health-enhancing physical activity. J Clin Epidemiol.

[29] Chinapaw MJM, Slootmaker SM, Schuit AJ, van Zuidam M, van Mechelen W (2009). Reliability and validity of the Activity Questionnaire for Adults and Adolescents (AQuAA). BMC Med Res Methodol.

[30] Owen N, Sugiyama T, Eakin EE, Gardiner PA, Tremblay MS, Sallis JF (2011). Adults’ sedentary behavior determinants and interventions. Am J Prev Med.

[31] Bland JM, Altman DG (1986). Statistical methods for assessing agreement between two methods of clinical measurement. Lancet.

[32] Bland JM, Altman DG (2010). Statistical methods for assessing agreement between two methods of clinical measurement. Int J Nurs Stud.

[33] McEneaney DF, Lennie SC (2011). Video instructions improve accuracy of self-measures of waist circumference compared with written instructions. Public Health Nutr.

[34] Rimm EB, Stampfer MJ, Colditz GA, Chute CG, Litin LB, Willett WC (1990). Validity of self-reported waist and hip circumferences in men and women. Epidemiology (Cambridge, Mass).

[35] Khunti K, Taub N, Webb D, Srinivasan B, Stockman J, Griffin SJ, Simmons RK, Davies MJ (2012). Validity of self-assessed waist circumference in a multi-ethnic UK population. Diabetic Med.

[36] Reidpath DD, Cheah JCH, Lam FC, Yasin S, Soyiri I, Allotey P (2013). Validity of self-measured waist and hip circumferences: results from a community study in Malaysia. Nutr J.

[37] National Institutes of Health (1998). Clinical Guidelines on the Identification, Evaluation, and Treatment of Overweight and Obesity in Adults--The Evidence Report. National Institutes of Health. Obes Res.

